# Migraine and Neuromodulation: A Literature Review

**DOI:** 10.7759/cureus.31223

**Published:** 2022-11-07

**Authors:** Varun Tiwari, Sachin Agrawal

**Affiliations:** 1 Department of Medicine, Jawaharlal Nehru Medical College, Datta Meghe Institute of Medical Science (Deemed to be university), Wardha, IND

**Keywords:** single-pulse transcranial magnetic stimulator, supraorbital neurostimulator, non-invasive vagus nerve stimulator, neuromodulation, migraine

## Abstract

Migraine is not only known to be one of the most common causes of a headache around the globe but is also the leading neurologic cause of disability worldwide. Migraine has significant social and economic effects. It not only hampers patients' quality of life but also hampers work, public conduct, and family life. Migraine is one of the leading causes of morbidity in the world, so effective management is critical. Currently, medical management is the mainstay remedial approach for migraine, but with time, non-pharmacological approaches, especially neuromodulation, are gaining popularity with a shred of solid backing evidence. Neuromodulation is the process in which specific devices are used to excite the central nervous system or peripheral nervous system with electric or magnetic, or any other form of energy to regulate the abnormal behavior of neural pathways that have occurred due to the disease process. Neuromodulation devices as approved by Food and Drug Administration include non-invasive Vagus nerve stimulators, single-pulse transcranial magnetic stimulators, and transcutaneous supraorbital neurostimulators. The purpose of this study is to summarize the information about the advances relating to neuromodulation concerning managing and preventing migraine. This Narrative review article is prepared after analyzing various research papers and publications on PubMed and Google Scholar. This article holds brief information on understanding neuromodulation, its mechanism, its implication in managing migraine, and its different modalities with their mechanism of action and contraindications. These neuromodulation techniques can certainly be used to deal with acute migraine attacks and inhibit their progression to chronic illness. Research is required on the application of neuromodulation in the early diagnosis of migraine, which is what we still lack as a whole medical fraternity.

## Introduction and background

Migraine is a syndrome of headache that occurs periodically and is accompanied by a wide range of symptoms of neurological impairment. It affects around 15% of females and 6% of males for one year [1]. The pathogenesis of migraine revolves around the trigeminal sensory pain pathways with the significant role of serotonin and dopamine. A migraine is an episodic headache associated with a wide range of clinical features and complications. There are various factors that act as triggers for causing an acute attack of migraine. With the advent of technology in the field of medicine, a revolution has set in, especially with respect to the diagnosis, treatment, and prevention of diseases. The same has also been achieved in the management of migraine. Now we have a wide range of pharmacological and non-pharmacological modalities for the treatment and prevention of migraine.

With the advent of biotechnology and biomedical engineering, medical science has achieved a new milestone in providing a non-pharmacological modality for the active management and prevention of migraine, known as Neuromodulation. It is an evolving treatment modality for dealing with a headache, directed as a non-invasive therapy for reducing pain by aiming at the constituents within the central and peripheral nervous system usually implicated in the pathophysiology of headaches, such as the sphenopalatine ganglion trigeminal nerve or vagus nerve for stimulation [2,3]. Currently, four Food and Drug Administration (FDA) approved neuromodulation devices are used in the market: Transcutaneous supraorbital neurostimulator, transcutaneous vagus nerve stimulator, single-pulse transcranial magnetic stimulator, and non-invasive multi-channel brain neuromodulation system.

Objectives

The primary objectives of conducting this study are to gather and summarize briefly the information about the etiology, pathogenesis, and clinical presentation of migraine; to study the currently used modalities for pharmacological management of migraine; to study the non-pharmacological modalities in the active management and prophylaxis of migraine; to collect information and present it systematically about the advances made in the field of neuromodulation concerning its application in migraine prophylaxis; to understand the future scopes and landmarks yet to be achieved concerning migraine; its etiology, pathogenesis, varying clinical presentations, and its management.

Methodology

This narrative literature review article is prepared after studying and analyzing various PubMed and Google Scholar publications. The keywords searched were migraine, neuromodulators, neurostimulators, transcutaneous supraorbital neurostimulator, non-invasive transcutaneous vagus nerve stimulator, and single-pulse transcranial magnetic stimulator. The study was restricted to literature in the English language, and the resultant articles were appraised based on their abstracts in order to embrace only the most relevant publications, dealing precisely with the pathophysiology behind migraine, clinical features of migraine, pharmacological and non-pharmacological management for migraine, advances in neuromodulation concerning treatment and prevention of migraine.

## Review

Migraine

The critical mechanism that underlies the generation of aches in migraine is hypothesized as the activation of trigeminal sensory pain pathways that occur due to the local widening of intracranial extracerebral blood vessels. This activation of the trigeminovascular system is believed to be the cause of the discharge of vasoactive sensory neuropeptides, particularly the Calcitonin gene-related peptide, which escalates the response to pain [4]. The contribution of serotonin, which is a 5-hydroxytryptamine neurotransmitter, is supported by evidence from various pharmacological and other data [5]. The role of dopamine is also backed by multiple studies in the pathogenesis of migraine [6]. The genetic aspect of migraine is recognized by reviewing families with Familial hemiplegic migraine (FHM). Mutations are known to occur in the SCN1A gene, which codes for voltage-gated sodium channels; ATP1A2 gene, which codes for Na+K+ATPase enzyme; and CACNA1A gene, which codes for voltage-gated calcium channels [7]. Migraine is an episodic headache disorder that is found to be linked with several combinations of neurologic, autonomic, and gastrointestinal symptoms [8]. The clinical course of Migraine is described in Table 1.

**Table 1:** Clinical course of migraine.

Phases	Clinical Features	Duration
Prodromal	Tiredness, Cognitive dysfunction, Yawning, Neck discomfort, Mood change, Food cravings, Polyuria.	Few hours to days.
Headache	Nausea, Photophobia, Phonophobia, Allodynia.	Few hours to days
Postdromal	Tiredness, Lack of concentration, Neck discomfort.	Few hours to days

Stage of chronic migraine sets in when patients have migraine episodes on eight or more days in a month and with no less than fifteen whole days of headache per month [9]. According to the current knowledge of migraine, patients with chronic migraine can suffer severe complications that may even prove fatal. These complications include migraine-triggered seizures, status migrainosus, persistent aura, and migrainous infarction. If not, migraine can also lead to other complications like depression, vertigo, anxiety, serotonin syndrome, sleeplessness, and rebound headaches.

Migraine can best be prevented by avoiding the triggers, but for that, the person should be well versed with the triggers that lead to his/her migraine attacks. There is a wide spectrum of triggers that can lead to an acute attack of migraine. These include lack of sleep, stress, certain food items, and hunger. Depending on the trigger, the relieving factors for migraine vary. For instance, a person suffering from a migraine due to lack of sleep feels better about taking a deep sleep, and a person suffering from a migraine due to stress feels better about getting relaxed. However, when the severity and frequency of migraine are critical, dealing with triggers cannot be the only solution, and this will require medical intervention. With time, as the world has learned more about the pathophysiology and clinical presentations of migraine, new pharmacological or non-pharmacological modalities have evolved for migraine management [10]. The medical management of migraine evolves mainly around the following classes of drugs mentioned in Table 2.

**Table 2:** Medical management of migraine.

Classes of Drugs	Drugs
Analgesics	Acetaminophen, Aspirin, Caffeine
Non-Steroidal Anti-Inflammatory Drugs	Naproxen, Ibuprofen, Tolfenamic acid
5-HT Receptor Agonists	Ergotamine, Sumatriptan, Frovatriptan, Almotriptan, Rizatriptan, Naratriptan, Zolmitriptan
Dopamine Receptor Antagonist	Metoclopramide, Chlorpromazine, Prochlorperazine

In the last five years or so, various trials have been conducted around the globe, and some new drugs have been learned to be efficient for the effective management of acute attacks of migraine, which include Lasmiditan (5-HT Receptor Agonist) [11] and Ubrogepant (C.G.R.P. Receptor Antagonist) [12]. Various classes of drugs have proved to be beneficial in the prevention of migraine and are used for the same [13]. These are mentioned in Table 3.

**Table 3:** Drugs for prevention of migraine.

Classes of Drugs	Drugs
Beta-blockers	Propranolol, Metoprolol
Anti-depressants	Amitriptyline, Dosulepin, Nortriptyline
Anti-convulsants	Topiramate, Valproate
Serotonergic Drugs	Pizotifen
CGRP Monoclonal Antibodies	Erenumab, Fremanezunab, Galcanezumab, Eptinezumab

Today in the era of technology, medical science is marching toward developing alternate therapeutic modalities over conventional pharmacological drug therapy for the management and prophylaxis of migraine and other headaches. The most rapidly growing modality is neuromodulation wherein with the application of different modes of stimulation, the activity of brain cells is altered.

Neuromodulation

Neuromodulation is the activation, regression, alteration, or modification of actions in the central or peripheral nervous system that employs electrical, magnetic, or chemical stimulation [14]. It involves aimed administration of a stimulus which can be chemical, electrical, or magnetic, to particular neural locations in the body for the function of moderation of nerve activity. Neuromodulation is characteristically a non-injurious, reversible, and adaptable process. It is becoming evident that this technology can treat migraines more effectively and safely than traditional medication therapies [15]. Clinicians and patients are becoming more interested in non-invasive neuromodulation therapies like non-invasive vagus nerve stimulation (nVNS) and single-pulse transcranial magnetic stimulation due to their proven effectiveness and safety [16,17]. As of today, it has been discovered that this technology offers enormous advantages, particularly for vulnerable patient populations like expectant mothers or those who have trouble tolerating medication or who experience its ineffectiveness. In certain situations, non-pharmacological neuromodulation techniques may be cost-effective [18].

Mechanism of Action

Neuromodulation functions by utilizing electrical or magnetic pulsations to influence or instead stimulate the central or peripheral pain pathways. This procedure seeks to control the pain systems to minimize the pain scores. Central neurotransmitters can be modified when pain circuits are stimulated electrically or magnetically [19]. For the treatment of acute migraine attacks, these variations possibly block the processes accountable for the generation of attack. For the purpose of prevention, these changes aim to reduce the central sensitization that leads to chronic headaches.

**Figure 1:**
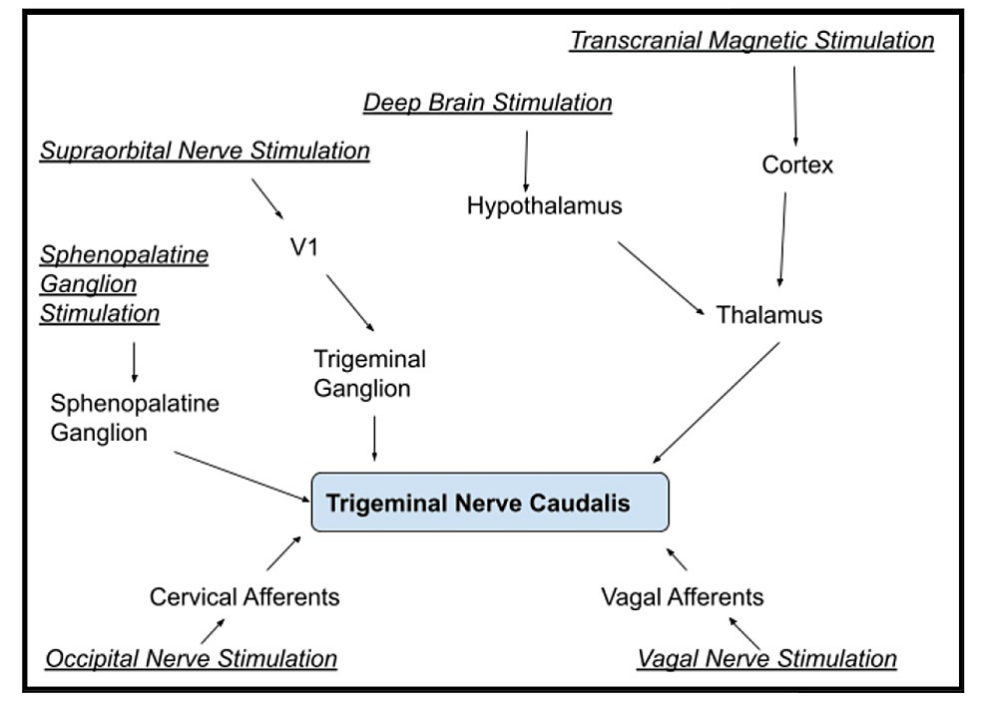
Neuromodulation techniques and their sites of action. Image credits: Varun Tiwari, Sachin Agrawal

The traditional methods stimulate the neurological system either centrally or peripherally through the skin, using either a variable magnetic field or an electric current to modulate the pain-related headache mechanisms. While both delivery methods have quick results, making them appropriate for treating acute symptoms, prolonged administration may have long-term preventative implications. [20]. Figure 1 shows various neuromodulation techniques with their respective site of action or targets.

Neuromodulation is carried out with a device that alters or adjusts the brain cell activity using electrical or magnetic stimulations. All such devices work; differently. Some of them stop attacks, while others are used preventatively. What is identical between them is the notion of altering the activity of the nerve pathways. Stimulators are another name for these instruments. Neuromodulation devices can be magnetic, electrical, or temperature changing. They can also be invasive or non-invasive, portable, or require surgical placement.

Neuromodulation modalities

**Figure 2:**
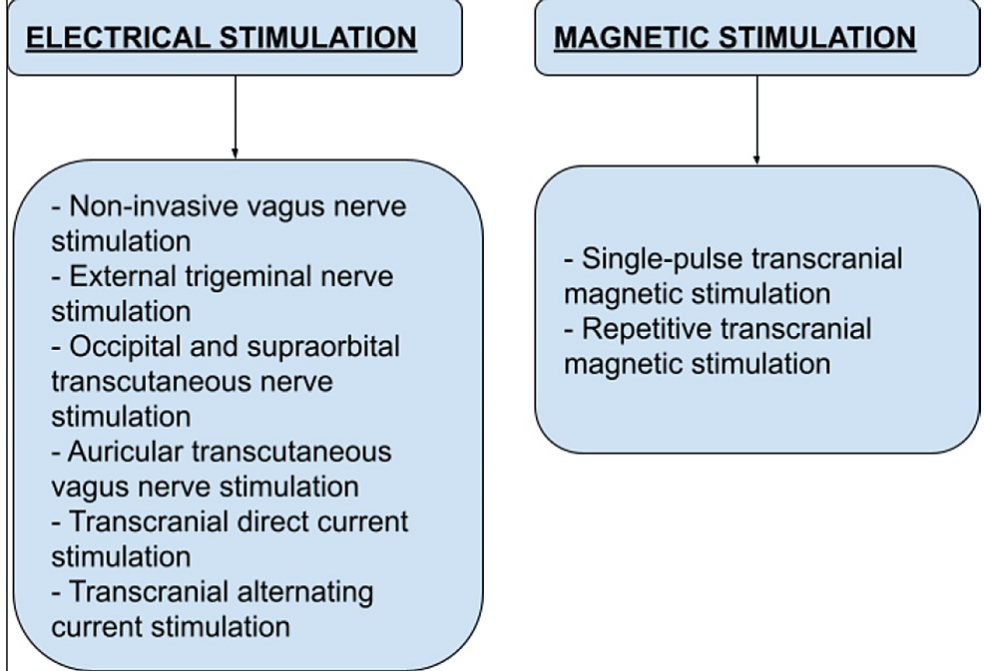
Non-invasive neuromodulations in the management of acute attacks of migraine. Image credits: Varun Tiwari, Sachin Agrawal

Since the advent of neuromodulation and its implication for prophylaxis of chronic pain conditions like migraine and cluster headache is known, various non-invasive neuromodulation modalities have been developed. Some of them have not only undergone the clinical trial phase but are also being used in the market. On the other hand, several neuromodulation techniques are currently undergoing clinical trials [21]. Figure 2 shows different non-invasive neuromodulations in the management of acute attacks of migraine.

Neuromodulation devices

Supraorbital nerve stimulators, non-invasive vagus nerve stimulators (nVNS), and Single-pulse transcranial magnetic stimulators (STMS) are among the most widely used neuromodulation devices approved by the FDA for management and prophylaxis-of-chronic headache disorders like migraine.

Single-Pulse Transcranial Magnetic Stimulator

The console device known as a single-pulse transcranial magnetic stimulator modifies the electrical signals in the brain by sending out a magnetic impulse. Previously it was exclusively licensed for treating acute migraine with aura, but more recently, it has also been approved to prevent migraines. In the realm of neuroscience, single-pulse transcranial magnetic stimulation has long been utilized as a safe, non-invasive method. To change the excitability of cortical neurons and thalamocortical circuits, it generates an oscillating magnetic field that, in turn, generates an electric current. Animal studies have demonstrated that STMS can reduce cortical spreading depression and the activation of nociceptive thalamic neurons that project to the cortex, which supports the growing interest in this method in the treatment of migraines [22].

The stimulation of meningeal nociceptors either directly or indirectly through descending assistance routes is reported to occur in migraine patients due to a state of brain hyperexcitability that lowers the threshold for cortical spreading depression, which in turn has been linked to the formation of aura in migraine patients [23,24]. Single-pulse transcranial magnetic stimulation can end an aura and diminish pain intensity in people who have an aura because it stops depression in the cortex from spreading. Repeated transcranial magnetic stimulation decreases hyperexcitability of the cortex by regulating glutamate and dopamine concentrations in the hippocampus and caudate. Therefore, persistent transcranial magnetic stimulation has the potential to alter neuronal excitability over the long term, reduce central sensitization, and reduce headache frequency. With the evidence of an improved benefit at twelve weeks compared with six weeks, patients should routinely utilize the instrument for at least three months before evaluating its effectiveness [25]. Clinical trials have shown that early management of migraine by single-pulse transcranial magnetic stimulator resulted in pain relief at two hours and a dearth of pain post-treatment was continued for 48 hours with no device-related adverse effects [26]. This device is now certified by FDA for the management and prevention of migraine in individuals 12 or more years of age. The dose for treatment of acute migraine attack comprises three consecutive pulses that can be given at the onset of an attack, followed by two pulses at an interval of 15 minutes, if required. The dose for prevention consists of four pulses that could be given twice daily [27].

Transcranial magnetic stimulators are absolutely not indicated in patients harboring active implantable instruments like defibrillators, pacemakers, cochlear implants, or any other electronic implantable devices which can be affected by a magnetic field. These devices are contraindicated for use in individuals who are known cases of epilepsy. These neuromodulators should be used cautiously in individuals with a positive history of cardiac diseases and disorders.

Non-Invasive Vagus Nerve Stimulator

The cervical branch of the 10th cranial nerve receives transcutaneous current from the gamma core device, a portable electrical stimulator. In the past, invasive vagus nerve stimulation (VNS) was indicated for treating depression and epilepsy. Interest in using VNS for managing headache problems was sparked by anecdotal narratives of migraine relief in individuals receiving implanted devices [28].

The limbic system, thalamus, nucleus tractus solitarius, dorsal pons, and locus coeruleus, all of which have been recognized in imaging studies as components of the headache pain composition, have been found to be suppressed by continuous VNS [29,30]. According to studies, it may lessen aches by lowering levels of glutamate in the trigeminal nucleus caudalis, which could reverse central sensitization in chronic headache sufferers [31]. Treatment focuses on the cervical branch of the tenth cranial nerve [32]. The randomized PRESTO study suggests the relieving effectiveness of a non-invasive vagus nerve stimulator between 30 to 60 minutes post a migraine attack. The study also found the effectiveness of this device in terms of pain relief, acceptability, and feasibility for the management of migraine [33].

Non-invasive vagus nerve stimulators are contraindicated for patients with active medical implants like pacemakers, defibrillators, cochlear implants, and any other electronic implantable devices near the treatment site. These simulators are also not to be used in individuals with a positive history of significant carotid atherosclerosis and cervical vagotomy. A vagus nerve stimulator should be used with extra precaution in case of vasovagal syncope and irritation of the skin near the site of treatment.

Supraorbital Nerve Stimulator

The Cefaly device was the first non-invasive neurostimulator licensed to treat migraines. A bipolar self-adhesive electrode (30 mm x 94 mm), when placed on the temple of the user of the Cefaly device, which is an external trigeminal nerve stimulation device, transcutaneously stimulates the supraorbital and supratrochlear branches of the ophthalmic nerve (V1) [34,35].

Supraorbital nerve stimulation causes an immediate sedative effect in healthy individuals [36]. Current knowledge of the pathogenesis of migraine points to neuronal hyperexcitability of the pain pathways as well as stimulation of the trigeminovascular system as the source of the phase of headache and central sensitization as the process contributing to the progression of migraine disease from intermittent to persistent. Supraorbital nerve activation possibly suppresses the trigeminal sensory pain routes by regulating both peripheral and central activity in the trigeminal-vascular system [37,38]. This device is less effective than topiramate at lowering migraine attacks. However, in topiramate trials, 50% of subjects experienced adverse effects related to the drug, and 25% of subjects stopped taking medication due to unacceptable negative symptoms [39]. These findings show that this device has a better efficiency/safety ratio and justifies being suggested for episodic migraine prevention before topiramate is prescribed [40].

These devices can cause sleepiness, headache, tingling sensations, and irritation of the skin after their use. The supraorbital nerve stimulator is not indicated for use in individuals with a recent brain or facial trauma within three months, in case of skin abrasions on the forehead near or in the area where electrodes are to be applied, in patients with allergy to acrylate. This technique of neuromodulation should be cautiously used in the case of electro-hypersensitivity.

## Conclusions

Migraine is one of the most intolerable types of headaches and one of the most common types of headaches throughout the world. It is a leading cause of morbidity and can also lead to certain fatal events in later stages, such as stroke. A lot has been achieved with respect to understanding the etiology, pathogenesis, and course of migraine. All the research to date has helped in the timely diagnosis of the disease, which is primarily clinical with limited use of investigatory biomarkers and management in the most appropriate way using both pharmacological and non-pharmacological modalities. A series of clinical trials have been taking place to provide a novel and more effective treatment and prevention approach to patients suffering from migraine. The use of neuromodulation is a big step in this direction. A neuromodulation is a non-pharmacological approach to dealing with patients with migraine that uses electric or magnetic stimulus to alter or modulate the central or peripheral nervous system. There are various devices that are used to stimulate nerve cells, which are called neuromodulator devices. Currently, three such devices are approved for use by FDA and are available on the market for the management and prophylaxis of migraine. These approved neuromodulator devices are non-invasive transcutaneous vagus nerve stimulators, transcutaneous supraorbital neurostimulators, single-pulse transcranial magnetic stimulators, and non-invasive multi-channel brain neuromodulation systems. These devices work on different mechanisms according to the pathogenesis of migraine. These devices must be used, keeping in mind their adverse effects and contraindications.

Thus, with strong supporting evidence, it can be summarized that non-invasive neuromodulation constitutes a practical approach to the management of migraine. However, the question that arises here is whether neuromodulation can be used as a modality for the diagnosis of migraine or not. There can be any application of neuromodulation in the investigatory profile of migraine or not. Nevertheless, in the future, this discipline will likely necessitate large-scale clinical trials that should result in a better knowledge of headache diseases like migraine as well as more individualized treatment methods.
